# Draft genome sequence of the moderately halophilic bacterium *Halobacillus* sp. BBL2006

**DOI:** 10.1016/j.dib.2018.11.076

**Published:** 2018-11-17

**Authors:** David S. Treves, James Francis, Gretchen Kirchner

**Affiliations:** Department of Biology, Indiana University Southeast, New Albany, IN, USA

## Abstract

We present the draft genome sequence of *Halobacillus* sp. BBL2006, a moderately halophilic, gram positive bacterium isolated from a sulfidic salt spring in Big Bone Lick State Park, Boone County, Kentucky. The genome of *Halobacillus* sp. BBL2006 was 3,988,138 bp in length with a GC content of 41.6%. Genome analysis identified 4331 open reading frames including genes for antibiotic resistance and tolerance to heavy metals. The draft genome was deposited at DDBJ/EMBL/GenBank (DNA Databank of Japan/European Molecular Biology Laboratory/Genbank) (JRNX00000000).

**Specifications table**

**Table:** 

Organism/cell line/tissue	*Halobacillus* sp.
Strain	BBL2006
Sequencer or array type	Illumina HiSeq
Data format	Analyzed
Experimental factors	Genomic DNA from bacterial pure culture
Experimental features	Isolation of bacteria, genome sequencing, draft genome assembly and
	annotation
Sample source location	Big Bone Lick State Park, Boone County, KY, USA
GPS coordinates	38.88690000, -84.74777778
Data accessibility	The draft genome was deposited at DDBJ/EMBL/GenBank under the accession number JRNX00000000 (https://www.ncbi.nlm.nih.gov/nuccore/JRNX00000000).

**Value of the data**

• These draft genome sequence data add to the growing but still limited list of *Halobacillus* genomes.
• *Halobacillus* isolates are of industrial importance for their production of halotolerant extracellular enzymes.
• These data provide a resource for studying gene structure and function in a salt-spring adapted *Halobacillus* isolate.

## 1 Data

The genus *Halobacillus*, proposed by Spring et al. in 1996 [1], contains isolates from diverse sources such salt lakes [1], [2], [3], solar salterns [4], [5], [6], [7], saline soil [8], [9], [10], deep sea methane seeps [11], sea anemone [12] and mangrove ecosystems [13], [14]. Many of this group׳s members are of industrial importance for their production of halotolerant extracellular enzymes [15], [16]. Noteworthy ecological functions of *halobacilli* include inhibition of quorum-sensing in gram negative bacteria [17] and production of compounds that stimulate plant growth [18].

**Fig. 1:**
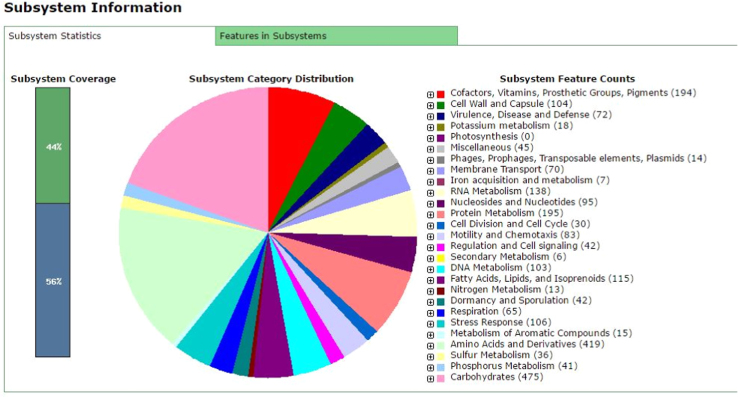
Subsystem distribution of *Halobacillus* sp. BBL2006 generated from the RAST annotation server.

*Halobacillus* sp. BBL2006 is a gram positive, moderately halophilic, endospore forming, light orange-yellow, rod shaped bacterium. De novo assembly of 1002 contigs with 84× coverage identified a genome size of 3,988,138 bp with a GC content of 41.6%. The NCBI (National Center for Biotechnology Information) Prokaryotic Genome Annotation Pipeline [19] documented 4331 genes including 3 rRNAs, 24 tRNAs and 184 pseudogenes. RAST (Rapid Annotation using Subsystem Technology) [20], [21] identified 446 subsystems including genes for heavy metal resistance (zinc, mercury, arsenic and cadmium) and genes for resistance to several classes of antibiotics (Fig. 1). In addition, a prophage was detected in the genome of BBL2006 which may assist in understanding gene transfer mechanisms in the *Halobacillus* genus. A BLAST (Basic Local Alignment Search Tool) query of Genbank using the 16S rRNA gene of BBL2006 identified *Halobacillus litoralis* (Accession no. NR_029304) [1], *Halobacillus trueperi* (Accession no. FJ937876) [22] and *Halobacillus karajensis* (Accession no. AJ486874) [8] as close relatives with 99% identity. This Whole Genome Shotgun project has been deposited at DDBJ/EMBL/GenBank under the accession JRNX00000000. The version described in this data article is version JRNX01000000.

## 2 Experimental design, materials and methods

*Halobacillus* sp. BBL2006 was isolated from a sulfur-enriched salt spring located in Big Bone Lick State Park, in Boone County, KY. BBL2006 genomic DNA was prepared using the Masterpure Gram positive DNA purification Kit (Epicentre) and purified genomic DNA was used for library construction and Illumina sequencing at htSEQ, Seattle, Washington, USA. BBL2006 was chosen for genome sequencing because its phenotypic features are representative of many *halobacilli* isolated from the Big Bone Lick State Park salt springs.
