# Supplementary material for: Draft genome sequence of the moderately halophilic bacterium Halobacillus sp. BBL2006
Source: Data Brief. 2018 Nov 17;21:2410–3. doi: 10.1016/j.dib.2018.11.076 (PMC6282633; doi:10.1016/j.dib.2018.11.076)
Supplement: Supplementary file 1 — Supplementary material [file mmc1.docx]

Conflict of Interest Statement

We as authors state that no conflict of interest exists.

We the authors have agreed to submit the attached article to Data in Brief.

The work described has not been published previously, it is not under consideration for publication elsewhere and publication is approved by all authors.
